# Stability of Saxitoxin in 50% Methanol Fecal Extracts and Raw Feces from Bowhead Whales (*Balaena mysticetus*)

**DOI:** 10.3390/md20090547

**Published:** 2022-08-25

**Authors:** Emily K. Bowers, Raphaela Stimmelmayr, Alicia Hendrix, Kathi A. Lefebvre

**Affiliations:** 1 Northwest Fisheries Science Center, Environmental and Fisheries Sciences Division, National Marine Fisheries Service, NOAA, 2725 Montlake Blvd E, Seattle, WA 98112, USA; 2 The North Slope Borough Department of Wildlife Management, P.O. Box 69, Utqiagvik, AK 99723, USA; 3 Department of Environmental and Occupational Health Sciences, University of Washington, Box 351618, Seattle, WA 98195, USA

**Keywords:** marine mammals, toxin degradation, harmful algal bloom toxins, storage conditions, ELISA, saxitoxin

## Abstract

In recent decades, harmful algal blooms (HABs) producing paralytic shellfish toxins (including saxitoxin, STX) have become increasingly frequent in the marine waters of Alaska, USA, subjecting Pacific Arctic and subarctic communities and wildlife to increased toxin exposure risks. Research on the risks of HAB toxin exposures to marine mammal health commonly relies on the sampling of marine mammal gastrointestinal (GI) contents to quantify HAB toxins, yet no studies have been published testing the stability of STX in marine mammal GI matrices. An understanding of STX stability in test matrices under storage and handling conditions is imperative to the integrity of toxin quantifications and conclusions drawn thereby. Here, STX stability is characterized in field-collected bowhead whale feces (stored raw in several treatments) and in fecal extracts (50% methanol, MeOH) over multiple time points. Toxin stability, as the percent of initial concentration (T0), was reported for each storage treatment and time point. STX was stable (mean 99% T0) in 50% MeOH extracts over the 8-week study period, and there was no significant difference in STX concentrations quantified in split fecal samples extracted in 80% ethanol (EtOH) and 50% MeOH. STX was also relatively stable in raw fecal material stored in the freezer (mean 94% T0) and the refrigerator (mean 93% T0) up to 8 weeks. STX degraded over time in the room-temperature dark, room-temperature light, and warm treatments to means of 48 ± 1.9, 38 ± 2.8, and 20 ± 0.7% T0, respectively, after 8 weeks (mean ± standard error; SE). Additional opportunistically analyzed samples frozen for ≤4.5 years also showed STX to be relatively stable (mean 97% T0). Mean percent of T0 was measured slightly above 100% in some extracts following some treatments, and (most notably) at some long-term frozen time points, likely due to evaporation from samples causing STX to concentrate, or variability between ELISA plates. Overall, these results suggest that long-term frozen storage of raw fecal samples and the analysis of extracts within 8 weeks of extraction in 50% MeOH is sufficient for obtaining accurate STX quantifications in marine mammal fecal material without concerns about significant degradation.

## 1. Introduction

In recent decades, paralytic shellfish toxins (PSTs) produced by harmful algal blooms (HABs) have become increasingly frequent in the marine waters of Alaska, USA [1]. PSTs are commonly produced by marine dinoflagellates of the genus *Alexandrium* and bioaccumulate in the marine food web when toxic *Alexandrium* cells are ingested by other organisms [2,3]. Saxitoxin (STX) is one of the most potent toxins among PSTs [4] and is the focus of this present study. 

As a potent neurotoxin, STX binds to and blocks voltage-gated sodium ion channels in muscle and nerve cells, causing an illness known as paralytic shellfish poisoning (PSP) in humans [5,6]. PSP is characterized by numbness, muscular weakness, sensations of “pins and needles” or floating, loss of coordination, drowsiness, difficulty breathing, and, in extreme cases, respiratory paralysis and death [7]. As the name suggests, PSP is most commonly contracted by eating PST-contaminated shellfish [4]. The first reported mass PSP event occurred in San Francisco, CA, in 1927, when 106 people became ill and 6 died after consuming contaminated shellfish [8]. More recently, nearly 2,000 human PSP cases are reported yearly worldwide, with annual mortality rates of about 15% [9].

STX has been detected in many trophic levels of Alaskan wildlife, including zooplankton, clams, worms, pelagic fishes, birds, and marine mammals, at higher latitudes than seen historically [3,10,11]. Additionally, the Chukchi Sea is home to one of the world’s most dense beds of *Alexandrium* cysts: algal cells transformed into a resting cyst life stage that lie dormant on the sea floor until certain environmental conditions are met, and then excyst into the vegetative cells that can form HABs [1,12,13]. As sea ice continues to recede and ocean temperatures rise, conditions are expected to become more favorable for HABs and *Alexandrium* cyst germination [14]. This may subject Pacific Arctic and subarctic coastal communities with limited or no prior HAB exposure to increased PSP risk, and subject wildlife, including marine mammals, to greater health threats [3,11,15,16].

Marine mammals, among other marine species, are critical to the food security, culture, and economy of Arctic and subarctic communities, and many local, tribal, academic, state, and federal institutions participate in the routine monitoring, health assessments, and research on the impact of HABs on their health. While little is known about the clinical effects of PSTs on marine mammals, PSTs have been associated with large-scale marine mammal mortality. They have been identified as the primary cause of death in a large humpback whale (*Megaptera novaeangliae*) die-off near Cape Cod in 1987 [17], implicated in the deaths of 117 Mediterranean monk seals (*Monachus monachus*) off the coast of the Western Sahara, Africa, in 1997 [18] and in the largest baleen whale (Sei whale, *Balaenoptera borealis*) mortality to date in Chile in 2015 [19], and are suspected to be a factor contributing to the endangerment of the Western North Atlantic right whale (*Eubalaena glacialis*) populations in the Bay of Fundy, Canada [20]. STX has been identified at sub-lethal concentrations in the gastrointestinal (GI) contents of 10 Arctic marine mammal species in a recent study including humpback whales, bowhead whales, and walruses [3], and was detected at moderate to high concentrations in a sample of 39 dead stranded walruses from the Bering Strait [1]. Given the changing environmental conditions, there are concerns that PSTs may begin appearing in concentrations harmful to Alaskan marine mammals in the near future, if not already.

Marine mammal GI contents are the primary matrices used in the detection and quantification of HAB toxins in marine mammals [3,20,21,22]. In order to assess the reliability of STX quantifications from these matrices, STX stability must be verified in raw GI material and in the extracts derived for analysis. From the literature, it is known that STX in algal extracts can be degraded by some bacteria of the genus *Pseudoalteromonas* found in the digestive tracts of blue mussels [23,24], and that less-toxic PSTs such as gonyautoxins or neo-saxitoxin can be biotransformed into STX within the body of a vector organism, increasing experienced toxicity [25]. STX is generally stable over time in low-pH, low-temperature storage conditions in matrices such as algae, algal extracts, and bivalves [26,27,28,29]. Commercial canning and some cooking practices have reduced PST toxicity by 50–70% in clams, mussels, and/or lobsters, though the reduction is generally attributed, at least partially, to toxin leaching and thus transfer to the cooking or packing media rather than toxin destruction [30,31,32,33]. However, there are no published studies exploring the stability of STX in marine mammal GI matrices. Delayed sample collection or improper storage is not uncommon for remote field sampling, bringing the reliability of subsequent toxin quantifications into question. Stability of STX during the collection and storage of these matrices is imperative to the integrity of toxin quantifications and subsequently drawn conclusions relating to wildlife and human health. This present study seeks to clarify the stability of STX in such matrices. 

The objective of this study was to characterize the stability of STX in field-collected marine mammal feces and fecal extracts stored under various conditions. STX concentration in bowhead whale (*Balaena mysticetus*) feces was quantified for two groups, similar to Bowers et al. [34]: (1) 50% MeOH extracts from feces; and (2) raw feces in various storage treatments (freezer, refrigerator, room-temperature (RT) light, RT dark, and warm incubator). Toxin stability, as the percent of initial concentration (T0), is reported here for each study group with statistical analyses employed when appropriate.

## 2. Results and Discussion

### 2.1. Extract Group

**Figure 1:**
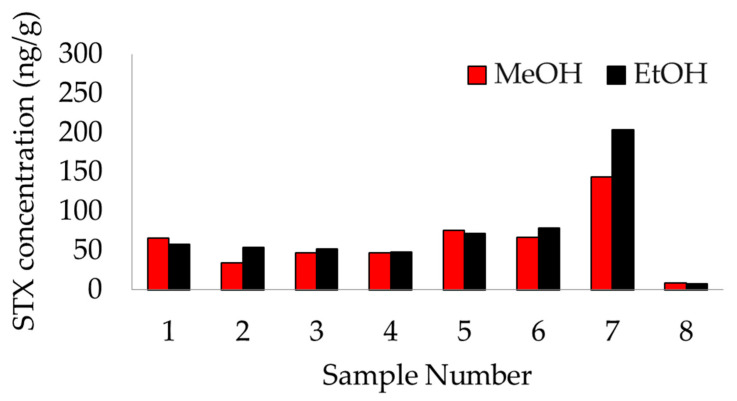
Results comparison between extraction solvents. Each sample was extracted using 80% ethanol (black) and 50% methanol (red). Results were quantified using the saxitoxin ELISA kit described in Section 3.4. A paired T-test found no statistically significant difference between extraction solvents.

To validate the extraction solvent, *n* = 8 marine mammal fecal samples were each extracted using 80% EtOH and 50% MeOH (Figure 1), and STX concentrations were compared between solvents for each sample. Toxin concentrations did not differ significantly between extraction solvents, verifying that 50% MeOH is an acceptable extraction solvent for STX quantification via ELISA compared to the more commonly used EtOH extraction protocol (referenced in Lefebvre et al. 2022).

**Figure 2:**
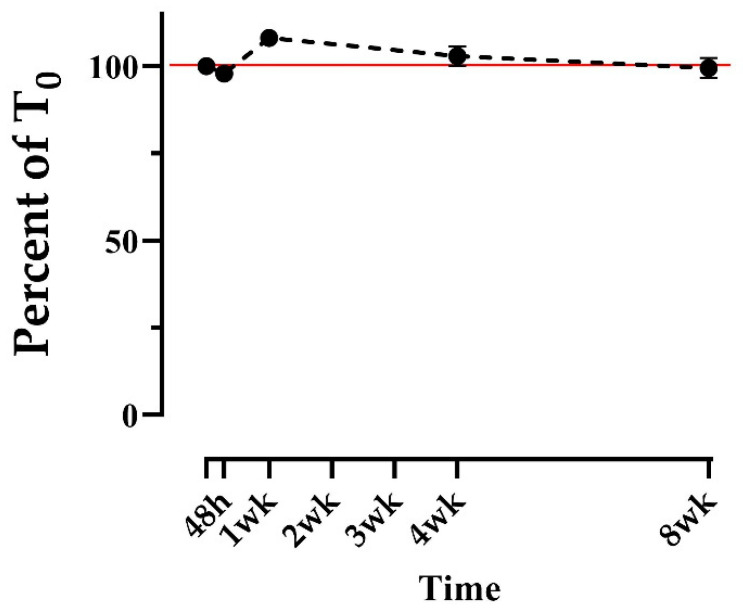
Stability of saxitoxin in 50% methanol extracts from bowhead whale feces over time, reported as the average of the percent of time zero concentration. Extracts were refrigerated at 1 °C in the dark. Each time point consists of the same *n* = 10 samples. A 100% reference line is included in red. Error bars display the standard error of the mean (SEM) and are too short to be depicted for the 48-hour, 1-week, and 2-week time points. No time points were significantly different from 100% of T0 except the 1-week point, which was significantly above T0 (*p* = 0.001).

STX was extracted using 50% MeOH from *n* = 10 bowhead whale fecal samples, and each extract was analyzed repeatedly throughout an 8-week study period to assess the stability of STX in the extracts. STX concentration as a percentage of T0 ranged from 97 to 108% on average, with no apparent trend (Figure 2). Standard errors of the mean (SEMs) remained low across all time points (<3%), likewise with no apparent trend. No time points were significantly different from 100% of T0 except the 1-week point, which was significantly above T0 (*p* = 0.001) at a mean of 108% (Figure 2). This increase was potentially due to variation in the ELISA plate on which those points were run, or human error during analysis. Regardless, these results suggest that STX extracted in 50% MeOH is stable for at least 8 weeks stored in a dark refrigerator at 1 °C. In contrast, the stability of domoic acid, another HAB neurotoxin, appeared to degrade by approximately 30% after 2 weeks in 50% MeOH extracts stored in the same condition [34].

### 2.2. Treatment Group

**Figure 3:**
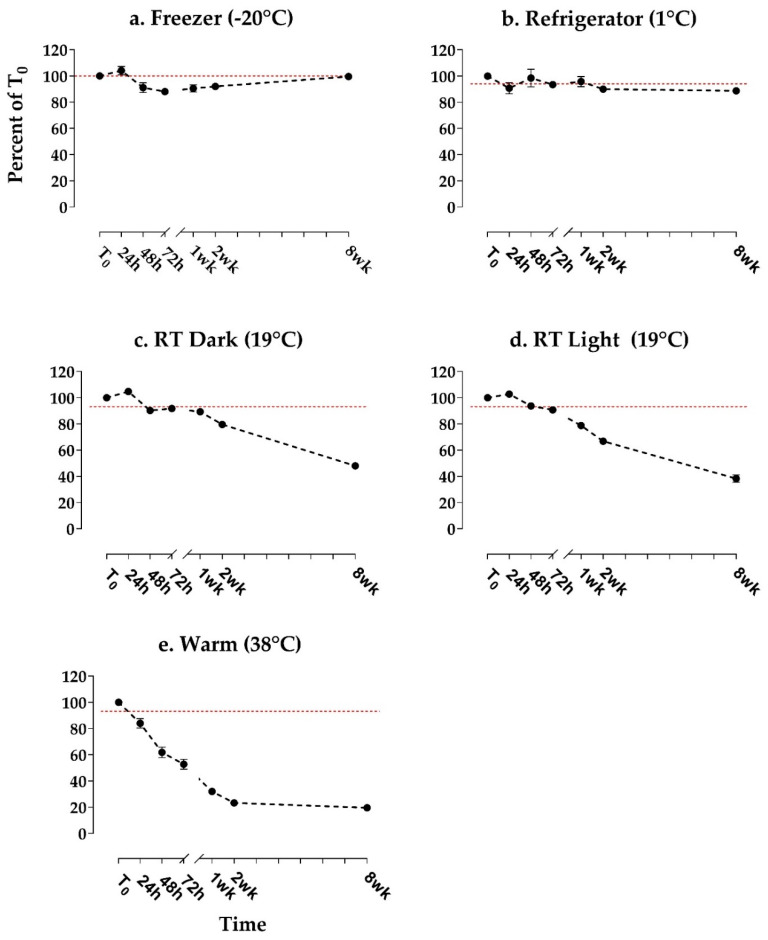
Stability of saxitoxin in raw bowhead whale feces over time, reported as the average of the percent of time zero concentration. Fecal aliquots were stored in 5 treatment conditions (**a**. freezer at −20 ˚C, **b**. refrigerator at 1 ˚C, **c**. room temperature (RT) dark at 19 ˚C, **d**. RT light at 19 ˚C, and **e**. incubator at 38 ˚C). Each time point consists of *n* = 4 samples. A 100% reference line is included in red. Error bars display the SEM.

Among the five treatment groups for raw feces storage, relative STX concentration remained constant in the freezer (−20 °C) and refrigerator (1 °C) treatments, but declined over time in the RT dark, RT light, and warm (38 °C) treatments (Figure 3). Average percent of T0 was measured between 88 and 104% (freezer) and between 89 and 99% (refrigerator) over the 8-week study period, with no apparent trend over time (Figure 3a,b). Conversely, the percent of T0 in the room-temperature treatments began declining slowly after 24–48 h, reaching an average of 48% (RT dark) and 38% (RT light) by week 8 (Figure 3c,d). Average percent of T0 in the warm treatment declined more quickly, being observed at 84% of T0 after just 24 h, decreasing steadily to an average of 23% after 2 weeks, then plateauing, being measured at 20% after 8 weeks (Figure 3e). SEMs were low (≤ 7%) for all treatments and time points (Figure 3). In contrast, domoic acid was found to be relatively stable across the same set of treatments in Bowers et al. [34].

These results suggest that STX is stable for ≤8 weeks in the freezer (−20 °C) and in the refrigerator (1 °C), but that it degrades over time in warmer conditions, degrading most rapidly in a warm incubator (38 °C). Interestingly, the warm treatment did not appear to continue degrading after 2 weeks. A longer follow-up experiment may be helpful in verifying this post-2-week plateau in the warm treatment, and for clarifying whether the percent of T0 would have also plateaued in the RT dark and light treatments, if given enough time. While the percent of T0 in the RT light treatment appeared to decline slightly more steeply than in the RT dark, the lack of statistical power for these data prevents us from drawing definitive conclusions regarding the impact of light on STX stability.

The slight increase above T0 observed in the freezer treatment after 24 h (Figure 3a) is likely due to moisture evaporation, causing STX to become more concentrated in those samples. Evaporation was also implicated when domoic acid measured above T0 in a similarly designed study by Bowers et al. [34], and by Smith et al. [35] in raw scallops. Using the same sample matrix and treatment conditions, Bowers et al. [34] reported more frequent and pronounced increases above T0 in domoic acid than seen in this present study. This difference may be due to less toxin degradation in all treatments in Bowers et al. [34], keeping relative toxin values closer to T0 and more likely to exceed it, or due to greater evaporation, as described above. Differences in evaporation between Bowers et al. [34] and this present study may be due to differences in sample handling between studies (sample tubes were opened once after being aliquoted in this present study, compared to several times in Bowers et al.). This suggests that reducing sample handling (e.g., opening of storage containers) may reduce moisture loss (and thus evaporation-driven increased toxin concentration) in a sample. 

### 2.3. Long-Term Frozen Group

**Figure 4:**
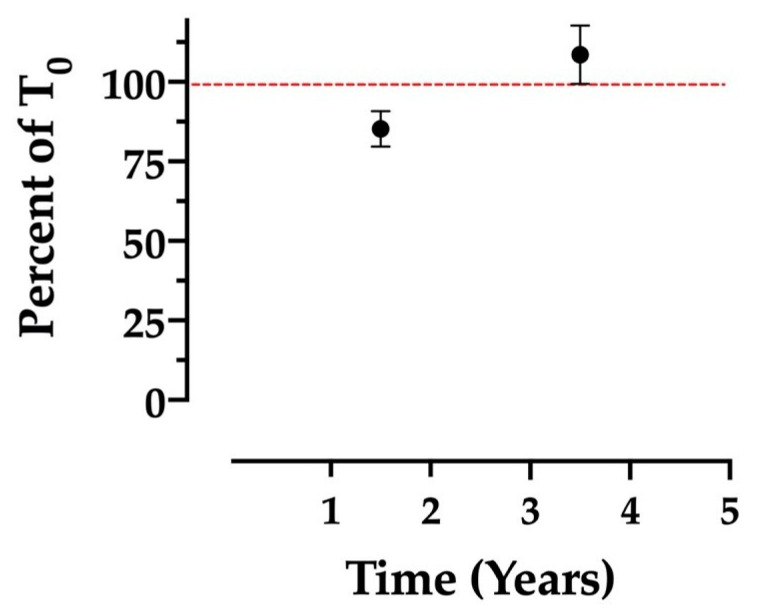
Stability of saxitoxin in raw, frozen bowhead whale feces over an extended time period, reported as the average of the percent of time zero concentration. Long-term time points consist of n=8 opportunistic samples, analyzed after 1.5 years (*n* = 4) and after 3.5 years (*n* = 4). Samples were each analyzed at only one time point. A 100% reference line is included in red. Error bars display the SEM.

Opportunistically analyzed fecal samples displayed relatively consistent STX concentrations after 1.5 and 3.5 years of freezer storage at −20 °C (Figure 4), though less consistent than the freezer and refrigerator treatments described above. The mean percent of T0 for samples analyzed after 1.5 years and 3.5 years was 85 and 108%, respectively, with respective SEMs of approximately 6 and 9% (Figure 4). The higher SEMs relative to the treatment groups are likely due to the opportunistic nature of the sampling, as storage conditions (container type, air space in container, etc.) were not necessarily uniform between samples. The increase above 100% of T0 observed in the 3.5-year time point may be attributable to moisture evaporation, as described above (2.2). Similar toxin concentration increases were observed for domoic acid in the opportunistic, long-term frozen samples studied in Bowers et al. [34]. Overall, however, the fact that STX concentrations stayed within 10% of their original values on average, following ≤3.5 years of freezer storage, suggests that STX can be considered sufficiently stable in raw feces stored frozen over long periods.

## 3. Materials and Methods

### 3.1. Sample Collection and Selection

Fecal samples used in this study were collected from subsistence-harvested bowhead whales in Utqiagvik, Alaska, USA in 2010–2021 (extract group), in 2017 (raw storage treatment group), and in 2008–2017 (opportunistic long-term frozen samples). Fecal material was removed from cut sections of colon using plastic spoons and stored in 50 mL polypropylene screw-cap tubes (Falcon-BD, Franklin Lakes, NJ, USA). Samples were frozen at −20 °C until analysis at the Northwest Fisheries Science Center’s Wildlife Algal-Toxin Research and Response Network (WARRN-West) laboratory (Seattle, WA, USA). STX was quantified in each sample prior to this present study. Leftover unanalyzed sample material remained frozen between analyses. Samples were selected for the present study based on their original STX concentrations (>100 ng/g).

### 3.2. Study Setup

The goal of the extract group was to characterize STX stability in the 50% MeOH extracts from marine mammal feces used for STX quantification and stored in the refrigerator in the dark, as is the authors’ standard laboratory procedure. Raw bowhead whale fecal samples (*n* = 10) were extracted following the extraction procedure outlined for the extract group below (3.3.1). Extracts were stored in a Kenmore top-mount refrigerator (1 °C; model no. 253.68972802).

The goal of the treatment group was to characterize STX stability over time in raw marine mammal feces under various controlled storage conditions. Fecal aliquots from *n* = 4 bowhead whales were stored under 5 treatments: freezer (−20 °C), refrigerator (1 °C), RT dark (19 °C), RT light (19 °C), and warm incubator (38 °C) (Table 1).

During the treatment period, freezer samples were stored in a Frigidaire freezer (model FFFU21M1QWE), and refrigerator samples were stored in the refrigerator specified above. All RT samples were kept on a north-facing window sill and RT light samples received natural (but not direct) sunlight during daylight hours in Seattle, WA, USA (February–April). RT light aliquot tubes were lined up on wire racks that allowed full light exposure, while RT dark samples were kept under a light-proof box. Ambient temperature was monitored for the RT samples using a TP-50 digital air thermometer (ThermoPro, Toronto, ON, Canada). Warm samples were kept in a Lab-Line (model no. 120) incubator, and internal incubator temperature was monitored using a Fisher USA thermometer (90 mm, Waltham, MA USA).

Additional raw fecal samples were also analyzed after longer periods of freezer storage to assess long-term STX stability under standard storage conditions (−20 °C; see freezer specifications above). Along with the four samples used in the 8-week short-term treatment study described above, eight additional raw fecal samples were analyzed following 1.5 years (*n* = 4) and 3.5 years (*n* = 4) of freezer storage. Each long-term sample was analyzed at only one time point.

### 3.3. Toxin Extraction

#### 3.3.1. Extract Group

Extraction was modified from the protocol established for STX extractions by Garthwaite et al. [36] to use MeOH rather than EtOH in the extraction solvent. To validate this adjustment, *n* = 8 marine mammal fecal samples (bowhead whale *B. mysticetus n* = 6, Pacific walrus *Odobenus rosmarus n* = 2) were each extracted twice, once using 80% EtOH and once using 50% MeOH. Extracts were analyzed via ELISA (see the STX ELISA kit described in Section 3.4). The resulting STX quantifications were compared for each animal using a paired T-test (α = 0.05). 

STX was extracted for the extract group samples using the method described in Bowers et al. [34]. Briefly, raw fecal samples were partially thawed and stirred thoroughly. Approximately 1 g of fecal material was aliquoted per sample. In total, 50% MeOH was added to each aliquot at 3× the aliquot weight for a 1-in-4 dilution. Sample solutions were vortexed, homogenized, and then centrifuged. Supernatants (the extracts) were collected and refrigerated until further analysis. Directly prior to toxin quantification, 200 μg subsamples of the extracts were filtered. Extracts were sampled, filtered, and re-analyzed at each time point.

#### 3.3.2. Treatment Group

Toxin extraction for the treatment (raw feces) group was performed as described above (Section 3.3.1), with slight modifications. Fecal samples were thawed in a small cooler, then refrigerated until aliquoted. Each sample was stirred, then aliquoted (0.25 g) into 1.5 mL microfuge tubes, making individual aliquots for every animal, treatment, and time point (4 animals × 5 treatments × 7 time points = 140 aliquots total). Samples were kept in the refrigerator until ready to aliquot and were aliquoted on ice. Light exposure was minimized during aliquoting. All aliquots were kept frozen for 24 h before the beginning of the study.

At each time point, one aliquot was extracted from each animal in each treatment. Aliquots were diluted using 50% MeOH as described above, then vortexed on high for 90 s. Any samples with remaining fecal clumps were vortexed for additional time, and/or clumps were broken using metal spatulas until samples were of uniform consistency. Finally, samples were incubated in the freezer for 5 min, then centrifuged (accuSpin Micro 17 Fisher Scientific, Waltham, MA USA) for 12 min at 12,000 rpm. Extracts (the supernatants) were poured off into 1.5 mL cryovials (220-3902-080, Evergreen Scientific, Caplugs CA, USA) and refrigerated until further analysis. Directly prior to toxin quantification, extracts were filtered as described above.

Long-term frozen treatment samples were aliquoted and extracted according to the procedure outlined for the extract group (Section 3.3.1), except long-term frozen samples were re-aliquoted and extracted at each time point.

### 3.4. Toxin Quantification

STX was quantified via direct-competition ELISA using commercially available Eurofins Abraxis Saxitoxin kits (Abraxis LLC, Warminster, PA, USA). These kits are designed to detect only STX, with limited cross-reactivity with other PSP toxins as described in the product documentation. Consequently, results are reported as STX equivalents and underestimate the potential impact of other congeners. Kits were used according to their manufacturer’s protocol with dilution modifications from Lefebvre et al. [3] (base dilution of 1:50 sample to 50% MeOH, plus any additional dilution necessary to ensure each concentration falls within the ELISA kit’s working range). Final dilution factors were determined individually, upon original analysis prior to this present study, and remained constant throughout the study. Loaded kit plates were incubated at room temperature on an orbital shaker (Bellco Biotechnology, Vineland, NJ, USA) for 30 min, then washed (ELx50, sn 257474, BioTek, Winooski, VT, USA). Kit-provided color solution was added to all wells before the plates were incubated again for 30 min on the orbital shaker. Well absorbance was quantified using a BioTek Epoch (sn 257814). The detection limit for samples by ELISA was 3 ng/g. STX concentrations quantified in all samples throughout this study ranged from 26 to 394 ng/g.

### 3.5. Data Analysis

STX concentrations were quantified using known standard absorbances and concentrations with the 4-parameter logistic curve fit model recommended in the ELISA kit protocol. Resulting concentrations were reported as percentages of the respective initial concentrations, T0 ([Ti/T0] × 100). For the extract and 8-week treatment groups, T0 was defined as the STX concentration quantified at the beginning of the present study. Conversely, the purpose of the long-term frozen samples was to assess STX stability in the years following original sample analysis; hence, T0 for long-term frozen samples was defined as the original concentration quantified years prior. All raw quantification results can be found in the Supplementary Materials published with this manuscript.

For the extract group, T-tests were used to assess differences between using 80% EtOH and 50% MeOH as extraction solvents (paired T-test; α = 0.05) and to determine whether the average %T0 at each time point was significantly different from 100% T0 (equal variance T-test; α = 0.05). Normality was assessed for both groups prior to the T-tests using the Shapiro–Wilk test. No statistical analyses were used to evaluate the treatment or long-term frozen groups due to small sample sizes (*n* = 4 replicates), which limited the reliability of data normality assessments and any subsequent parametric or non-parametric analyses [37,38,39]. Sample size was constrained in this study by sample availability, sample volume, and original toxin concentration.

## 4. Conclusions

Evidence points to a global increase in HAB toxin events and geographic range, posing an increasing threat to marine mammals and their ecosystems. It is important that the methods by which HAB toxins are quantified are reliable and accurate, to support the usefulness of research on the ecological impacts of such toxins. This present study confirmed that 50% MeOH is an appropriate extraction solvent for marine mammal feces, yielding results comparable to the previously established 80% EtOH extraction solvent. STX in 50% MeOH extracts from bowhead whale feces was shown to remain stable (mean 99% of T0) after 8 weeks, with extracts stored in the refrigerator (1 °C) in the dark. Results from raw feces stored in various treatments suggested that keeping samples frozen (−20 °C) was the most appropriate storage condition for preserving STX therein, though refrigerator storage (1 °C) up to 8 weeks appeared to be an equally valid short-term storage option, if necessary. In this treatment group, STX was observed degrading over time when exposed to sunlight and warm temperatures. STX in long-term frozen samples also appeared relatively stable, remaining near 100% of T0 (mean 85% after 1.5 years, 108% after 3.5 years). Increases above 100% of T0 likely occurred due to moisture evaporation from the long-term storage samples, causing STX to concentrate in the remaining material. Comparing both the apparent effects of evaporation and sample storage methods between this present study and Bowers et al. [34], it appears that reducing sample handling (e.g., opening sample containers, re-aliquoting) may reduce the risk of evaporation from samples. Additional variability may also be introduced by differences between ELISA plates and by human error in analysis execution. Apart from Bowers et al., [34], there are no other published studies regarding the stability of algal toxins in marine mammal GI matrices to which this present study can be compared. Overall, these results serve to validate the methods by which marine mammal fecal samples are handled prior to HAB toxin quantification, enabling rigorous scientific assessments of HAB toxins and their potential effects on marine wildlife.

## Figures and Tables

**Table 1:** Summary of treatment group design and justification. Based on Bowers et al. [34].

Treatment Name	Temperature	Justification
Freezer	−20 °C	Ideal condition in which samples are stored immediately upon collection
Refrigerator	1 °C	Second best storage option if freezing is not possible
Room Temperature (RT)—Dark	19 °C ± 2 °C ^1^	Accidental/unavoidable exposure to ambient temperatures (e.g., field, laboratory) in the dark
Room Temperature (RT)—Light	19 °C ± 2 °C ^1^	Accidental/unavoidable exposure to ambient temperatures (e.g., field, laboratory) in daylight
Warm	38 °C ± 1 °C ^1^	Delay of sample collection from a dead carcass

^1^ Mean temperature ± standard deviation.

## Data Availability

The data presented in this study are available as Supplementary Material published with this manuscript.

## Supplementary Materials

The following supporting information can be downloaded at: https://www.mdpi.com/article/10.3390/md20090547/s1. These include analysis dates, dilution factors, and raw STX quantification results for each sample analyzed in the extract, treatment, opportunistic long-term frozen, and extraction solvent comparison groups.
